# Supplementary material for: Chaperone-mediated ordered assembly of the SAGA and NuA4 transcription co-activator complexes in yeast
Source: Nat Commun. 2019 Nov 20;10:5237. doi: 10.1038/s41467-019-13243-w (PMC6868236; doi:10.1038/s41467-019-13243-w)
Supplement: Supplementary file 1 — Supplementary Information [file 41467_2019_13243_MOESM1_ESM.pdf]

Supplementary information for:

Chaperone-mediated ordered assembly of the SAGA and NuA4 transcription co-activator complexes in yeast

Alberto Elías-Villalobos *et al*.

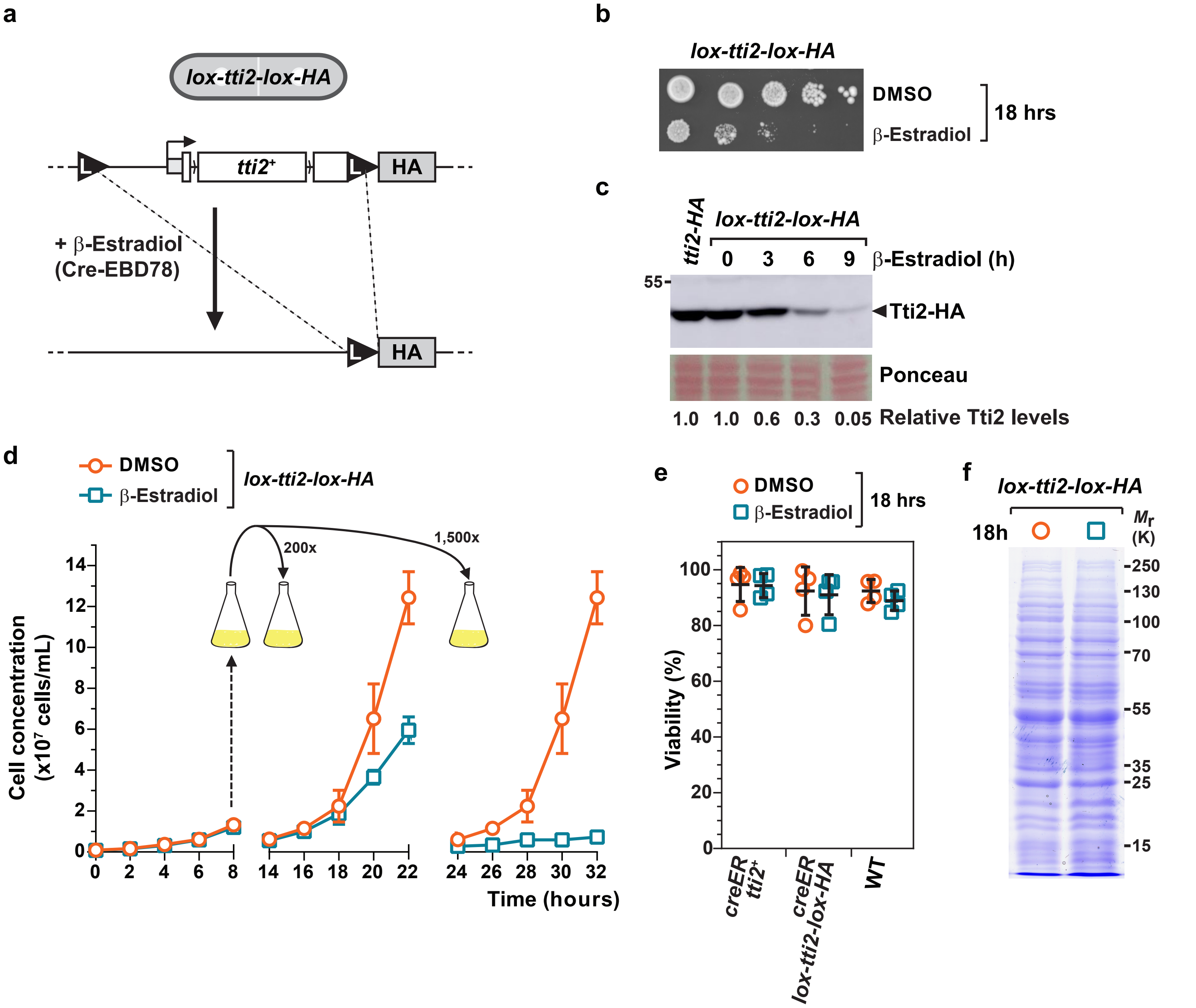

Supplementary Figure 1. CreER-mediated inducible knock-out of Tti2 (*tti2-CKO* strains).
a Illustration of the *tti2*+ locus engineered with loxP sites (L black triangles) flanking the entire gene, before and after β-estradiol-induced Cre recombinase activity.
b Estimation of *lox-tti2-lox* locus recombination efficiency. Ten-fold serial dilutions of *tti2-CKO* cultures treated with either DMSO or β-estradiol for 18 hours were spotted on rich medium and incubated for 3 days at 32°C.
c Anti-HA Western blot analysis of Tti2-HA expression at different time points upon *tti2*+ knock-out, as compared to a non-loxed *tti2-HA* strain. Ponceau red staining is used as a loading control. Shown below is a quantification of Tti2-HA signal intensity from linear chemiluminescence exposures, normalized to Ponceau red signals, and averaged from two independent experiments (n = 2).
d Proliferation defects of *tti2-CKO* strains estimated from growth curves of liquid cultures diluted such as to count cells during exponential growth, at different time points, in DMSO- and β-estradiol-containing media (orange circles and blue squares, respectively). Each value represents the average number of cells from four independent replicates with the SD (n = 4). At least 50 cells from the indicated genotypes were counted at each time point.
e Viability of *tti2-CKO* strains grown in liquid media and treated with either DMSO (orange circles) or β-estradiol (blue squares) for 18 hours. The percentage of viable cells was estimated from methylene blue staining of liquid cultures and counting at least 200 cells by light microscopy. Four independent experiments are shown as individual points, overlaid with the mean and SD (n = 4).
f Coomassie blue staining of total protein extracts from *tti2-CKO* strains grown as in (**e**).
Source data are provided as a Source Data file.

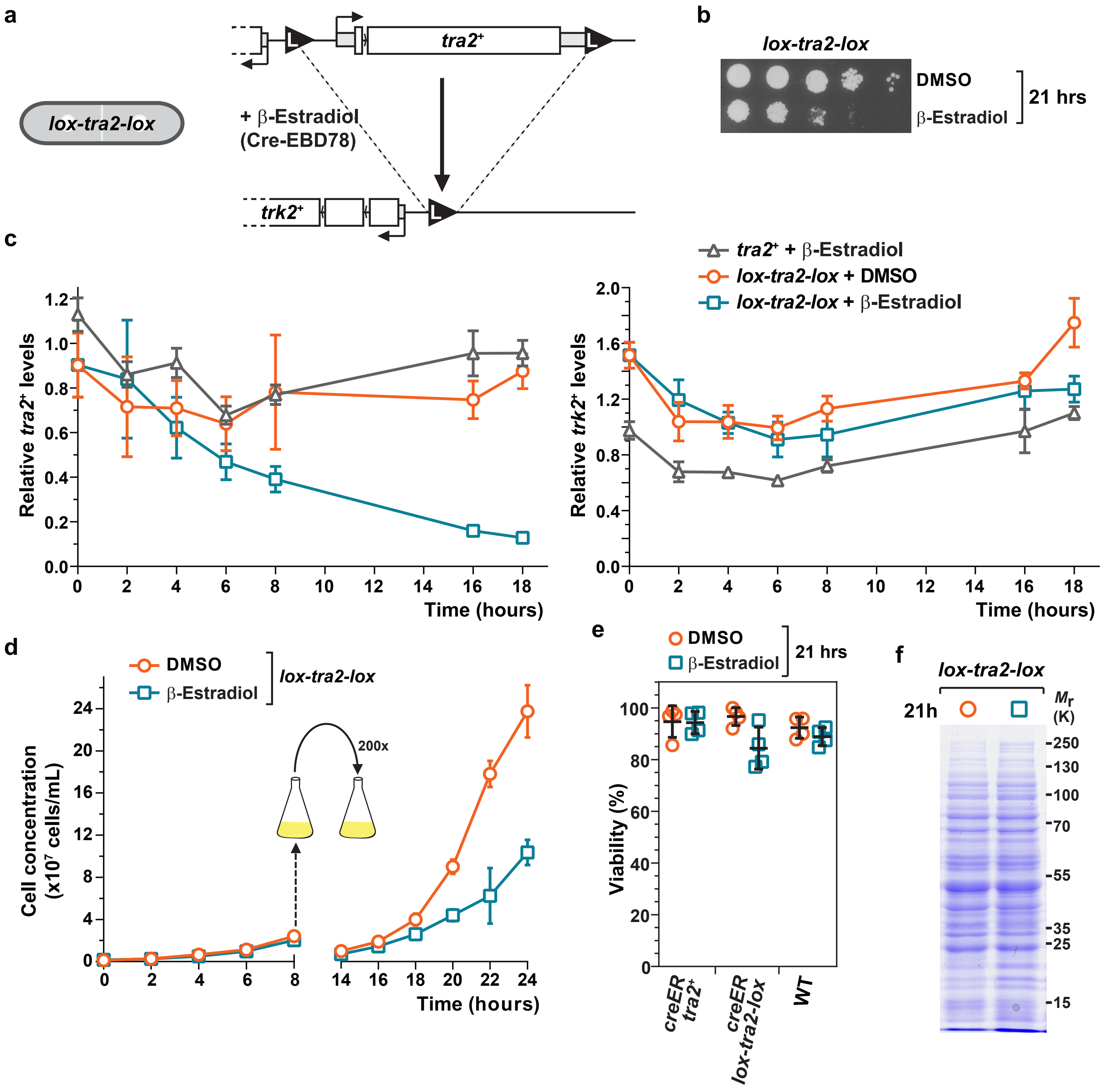

Supplementary Figure 2. CreER-mediated inducible knock-out of Tra2 (*tra2-CKO* strains).

a Illustration of the *tra2*+ locus engineered with loxP sites (L black triangles) flanking the entire gene, before and after β-estradiol-induced Cre recombinase activity.

b Estimation of *lox-tra2-lox* locus recombination efficiency. Ten-fold serial dilutions of *tra2-CKO* cultures treated with either DMSO or β-estradiol for 21 hours were spotted on rich medium and incubated for 3 days at 32°C.

c RT-qPCR analysis of the expression of *tra2*+ (left) and of its divergently transcribed 5' neighbouring gene, *trk2*+ (right), at different time points upon *tra2*+ knock-out. A wild-type control strain grown in β-estradiol (grey triangles) and a *tra2-CKO* strain grown either in DMSO (orange circles) or in β-estradiol (blue squares) were analyzed. Each value represents mean mRNA levels from four independent RT-qPCR experiments with the SD (n = 4). *act1*+ served as a control for normalization across samples. Values from one control experiment were set to 1 to allow comparisons across culture conditions and mutant strains.

d Proliferation defects of *tra2-CKO* strains estimated from growth curves of liquid cultures diluted such as to count cells during exponential growth, at different time points, in DMSO- and β-estradiol-containing media (orange circles and blue squares, respectively). Each value represents the average number of cells from four independent replicates with the SD (n = 4). At least 50 cells from the indicated genotypes were counted at each time point.

e Viability of *tra2-CKO* strains grown in liquid media and treated with either DMSO (orange circles) or β-estradiol (blue squares) for 21 hours. The percentage of viable cells was estimated from methylene blue staining of liquid cultures and counting at least 200 cells by light microscopy. Four independent experiments are shown as individual points, overlaid with the mean and SD (n = 4).

f Coomassie blue staining of total protein extracts from *tra2-CKO* strains grown as in (**e**).

Source data are provided as a Source Data file.

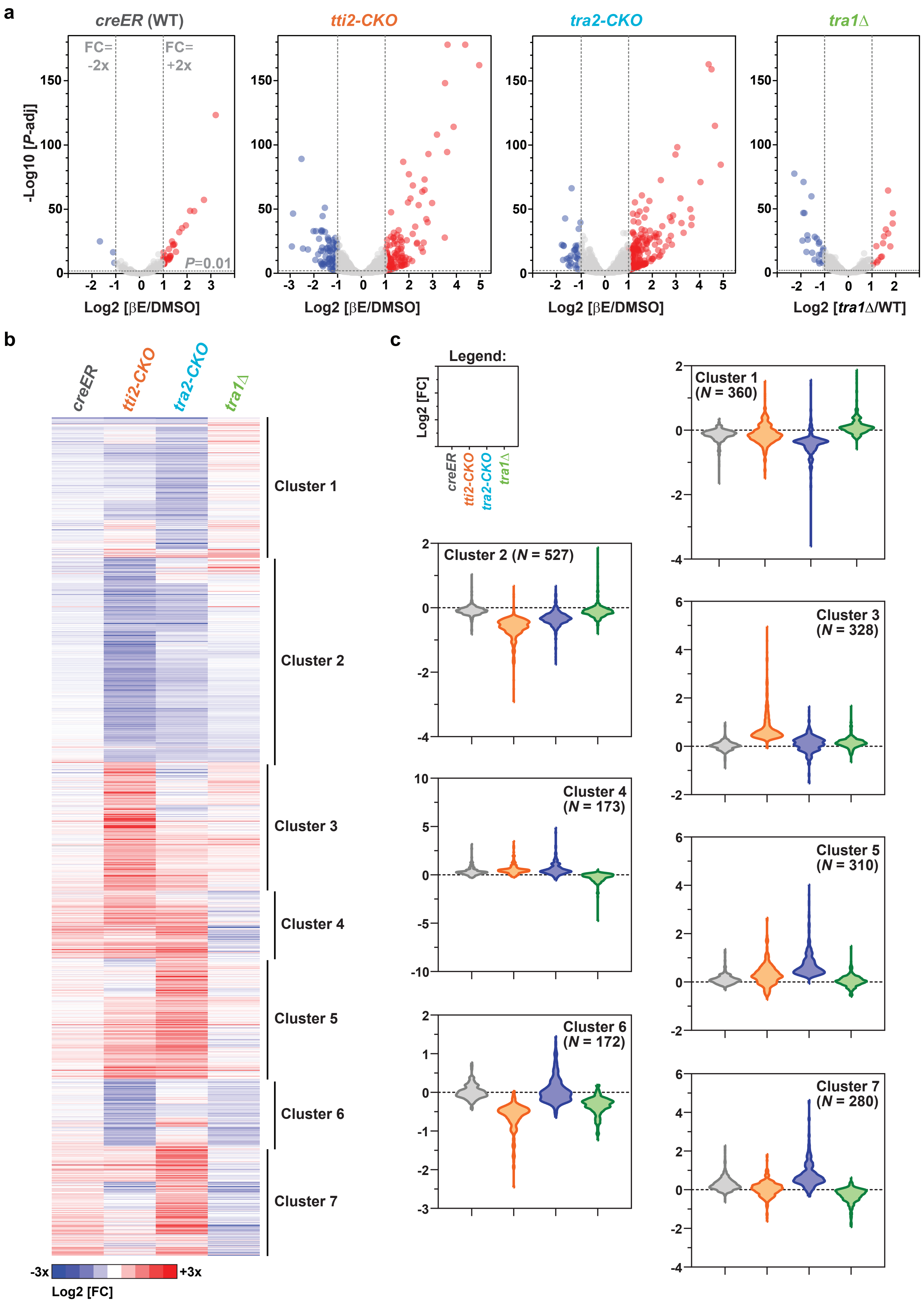

Supplementary Figure 3. Transcriptome analyses of *tti2-CKO*, *tra2-CKO* and *tra1*Δ mutants. *(See next page)*

Supplementary Figure 3. Transcriptome analyses of *tti2-CKO*, *tra2-CKO* and *tra1*Δ mutants.
a Volcano plots of RNA-seq data comparing the fold change of normalized counts *versus* the adjusted *P* value, calculated using the DESeq2 package. RNA-seq were performed in triplicate from control *creER* (WT), inducible *tti2*+ knock-out (*tti2-CKO*), inducible *tra2*+ knock-out (*tra2-CKO*), and *tra1*Δ mutants. Differential gene expression analysis was performed by comparing cells treated with DMSO and β-estradiol (βE), for either 21 hours (*creER* and *tra2-CKO* strains) or 18 hours (*tti2-CKO* strain). *tra1*Δ mutants were compared to isogenic wild-type (WT) control cells. Up- and down-regulated genes with a fold change ≥ 2 and a *P* value ≤ 0.01 are coloured in red and blue, respectively, and these thresholds are shown as grey dashed lines.
b Heatmap of differentially expressed genes ordered by clusters from RNA-seq data of control *creER* (WT), inducible *tti2*+ (*tti2-CKO*) and *tra2*+ knock-outs (*tra2-CKO*), and *tra1*Δ mutants (columns). Differential gene expression analysis was performed as in (**a**). Rows represent genes that are differentially expressed in at least one condition (*P* ≤ 0.01), with their fold change (Log2 [FC]) colour-coded as indicated in the scale bar. Hierarchical clustering of genes was performed in Cluster 3.0, using the complete linkage method and based on Pearson correlation distance measures, and revealed seven major clusters.
c Violin plots showing the distribution of gene expression changes within each of the seven clusters identified in (**b**). In each graph, Log2 fold changes are shown (Log2 [FC]) in control strains (*creER*, grey), upon depletion of Tti2 (*tti2-CKO*, orange), of Tra2 (*tra2-CKO*, blue), and upon Tra1 deletion (*tra1*Δ, green).
Source data are provided as a Source Data file.

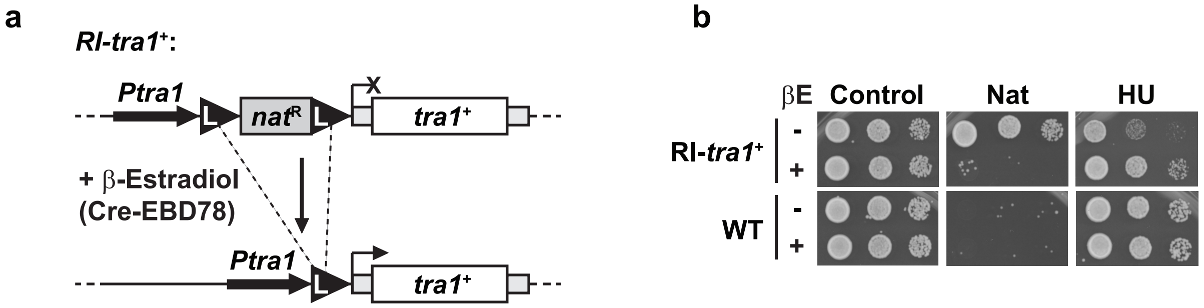

Supplementary Figure 4. Genetic assay to follow the fate of Tra1 upon *de novo* synthesis.

a Illustration of the strategy to follow the fate of newly synthesized Tra1 (Recombination Induced-*tra1*+). A strong transcription terminator sequence from the *Ashbya gossypii TEF1* gene, included in the antibiotic resistance cassette, flanked by two *loxP* sites (L black triangles) was inserted between *tra1*+ promoter and ORF. Addition of β-estradiol allows recombination-induced *de novo* expression of Tra1 at endogenous levels.

b Growth phenotype of control WT and *RI-tra1*+ strains, grown in liquid media and treated with either DMSO (-) or β-estradiol (+). Ten-fold serial dilutions were spotted on rich medium (control), supplemented with Nourseothricin (Nat) or 10 mM hydroxyurea (HU), and incubated for 3 days at 32°C.

Source data are provided as a Source Data file.

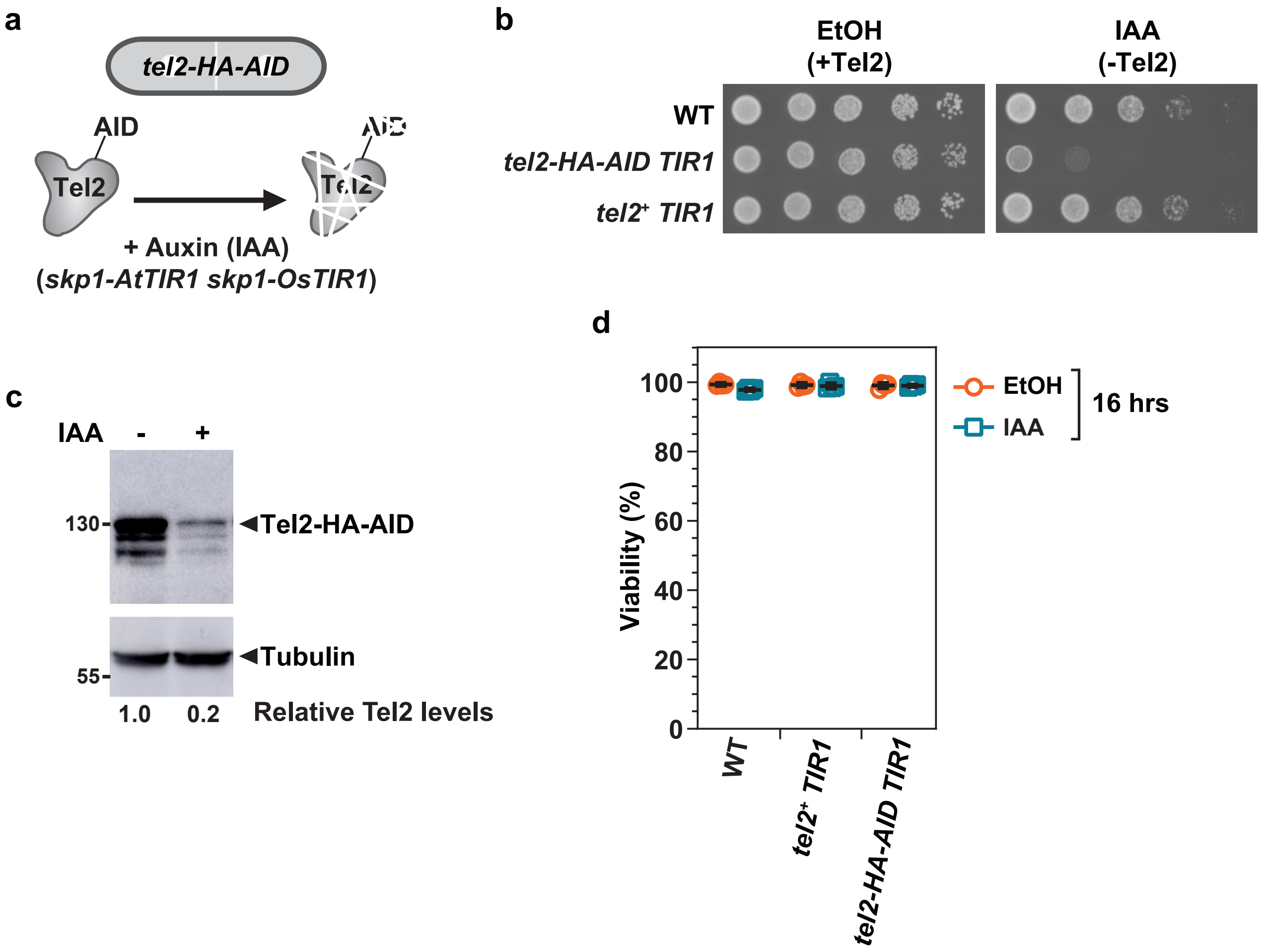

Supplementary Figure 5. Auxin-mediated inducible depletion of Tel2.

a Illustration of the auxin-inducible degron (AID) system for endogenous Tel2 depletion, using strains in which *S. pombe* Skp1 is fused to *Arabidopsis thaliana* Tir1 and *Oryza sativa* Tir1. Addition of the plant hormone auxin to the media (IAA; indolacetic acid) allows the rapid and conditional degradation of Tel2.

b Growth phenotype cells depleted for Tel2. *tel2-HA-AID skp1-AtTIR1 skp1-OsTIR1* strains were grown to exponential phase in minimal medium supplemented with either ethanol (EtOH; +Tel2) or auxin (IAA; -Tel2). Ten-fold serial dilutions were then spotted on corresponding solid media and grown for 4 days at 25°C. WT and *skp1-AtTIR1 skp1-OsTIR1* strains were used as controls.

c Anti-HA Western blot analysis of Tel2-HA-AID expression 16 hours after adding auxin. An anti-tubulin antibody served as loading control. Shown below is a quantification of Tel2-HA signal intensity from linear chemiluminescence exposures, normalized to Tubulin signals, and averaged from two independent experiments (n= 2).

d Percentage of viable cells estimated from methylene blue staining of liquid cultures and counting at least 200 cells by light microscopy. Four independent experiments are shown as individual points, overlaid with the mean and SD (n = 4).

Source data are provided as a Source Data file.

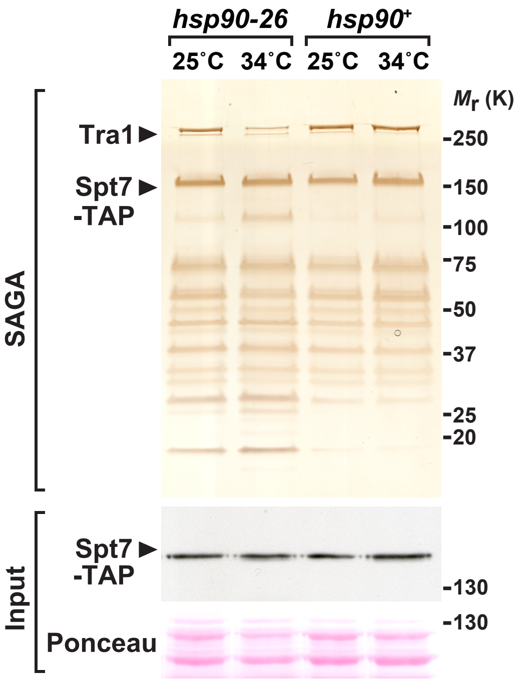

Supplementary Figure 6. Hsp90 promotes Tra1 incorporation into the SAGA complex.

Silver staining analysis of SAGA complexes purified from WT and *hsp90-26* mutants, using Spt7 as the bait. Cells were grown to exponential phase at the permissive temperature (25˚C) and shifted for 6 hours at the restrictive temperature (34˚C). Below is an anti-HA Western blot of Spt7-TAP from a fraction of the input used for TAP. Ponceau red staining is used as loading control. Shown are data that are representative of two independent experiments. Source data are provided as a Source Data file.

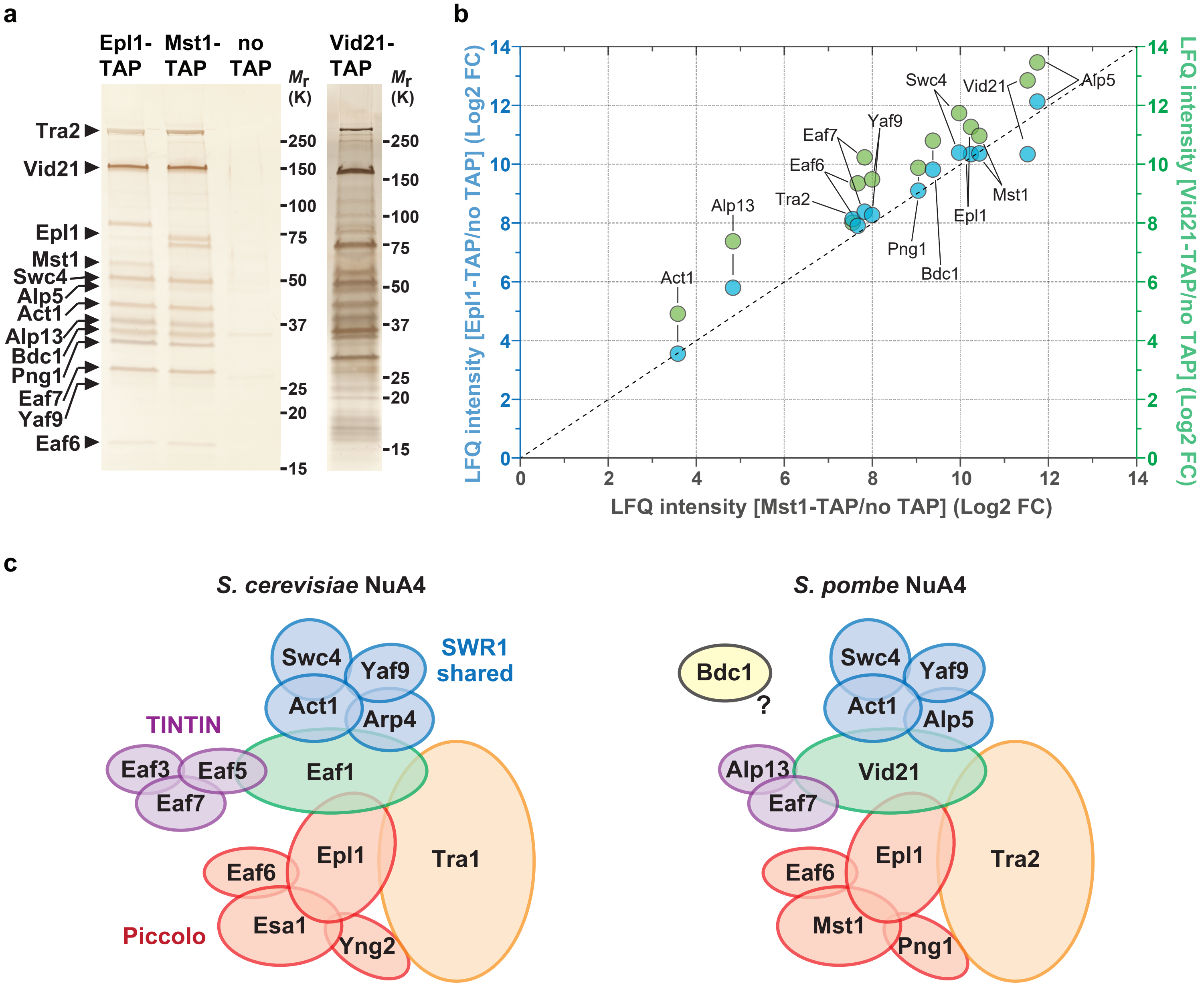

Supplementary Figure 7. Characterization of the *S. pombe* NuA4 complex.

a Silver staining analysis of NuA4 complexes purified using Mst1, Epl1, or Vid21 as baits. A non-tagged strain (no TAP) was used as a control for background. The position of each NuA4 subunit is annotated on the gel according to their predicted molecular weight.

b Tandem mass spectrometry analyses (LC-MS/MS) of tandem affinity purified Mst1, Epl1, and Vid21 from (**a**). Each individual point represents the LFQ intensity ratio of a protein in TAP eluates relative to a ‘no TAP’ control. For Epl1-TAP and Vid21-TAP, each value represents an average from two independent replicates (n = 2). Blue compares Epl1 with Mst1 eluates, whereas green compares Vid21 with Mst1 eluates. The black dashed line represents a 1:1 ratio.

c Comparison of NuA4 complex subunit composition between *S. cerevisiae* and *S. pombe*.

Source data are provided as a Source Data file.

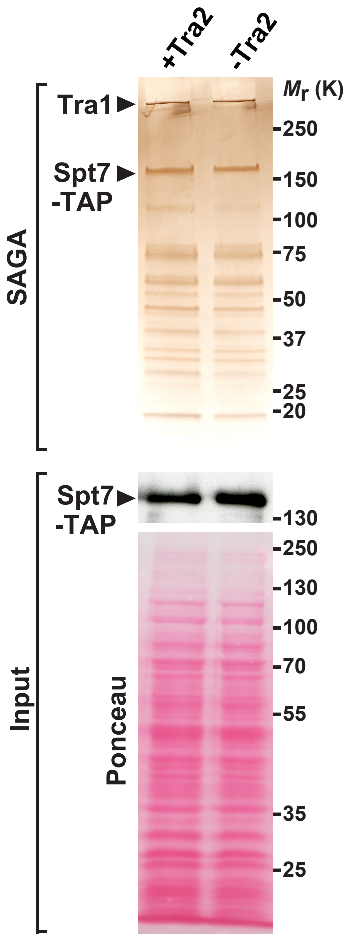

Supplementary Figure 8. Control SAGA purifications upon conditional depletion of Tra2.

Silver staining of SAGA complexes purified either in presence or absence of Tra2, using Spt7-TAP as the bait. *spt7-TAP tra2-CKO* cells were grown to exponential phase in rich medium supplemented with either DMSO (+Tra2) or β-estradiol (-Tra2) for 21 hours. Below is an anti-HA Western blot of Spt7-TAP from a fraction of the input used for TAP. Ponceau red staining is used as loading control. Shown are data that are representative of two independent experiments. Source data are provided as a Source Data file.

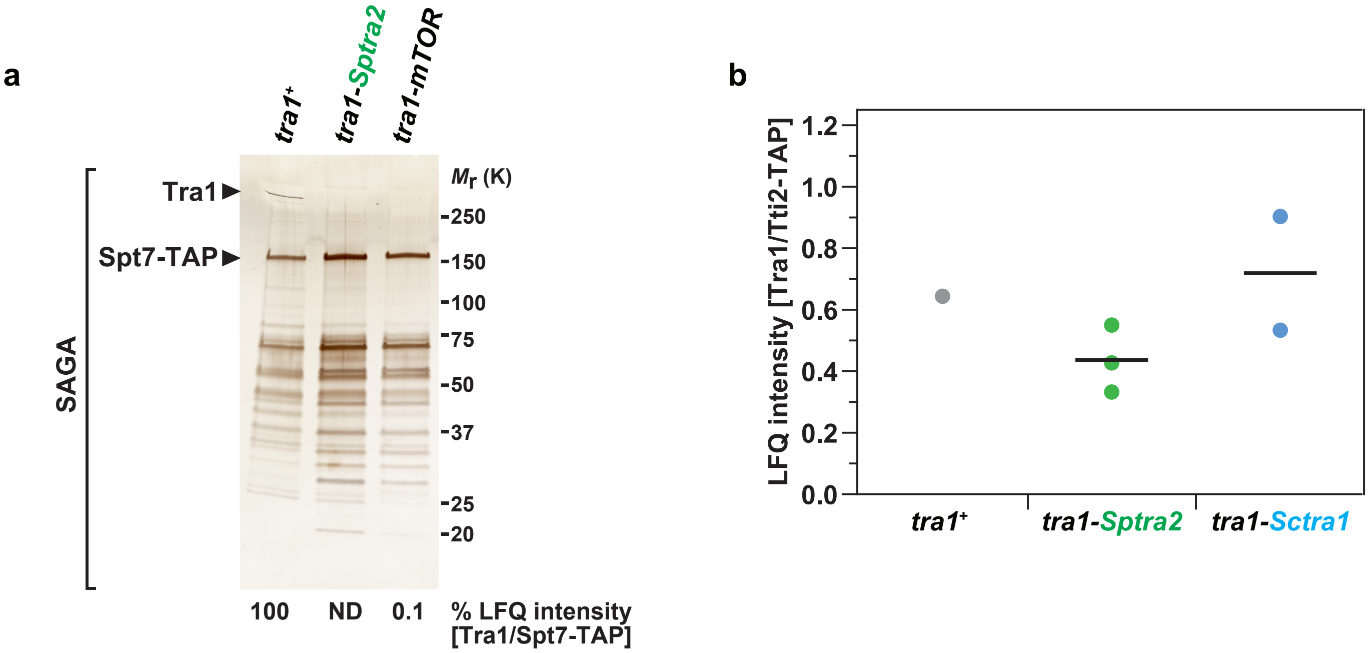

Supplementary Figure 9. The CSI region of Tra1 is not required for its interaction with TTT.

a Residues 2623-2676 from *S. pombe* Tra1 were swapped with the homologous region from either *S. pombe* Tra2 (residues 2564-2617, green), to create the *tra1-Sptra2* allele, or *Homo sapiens* mTOR (residues 1456-1504), to create the *tra1-mTOR* allele. Silver staining analysis of SAGA complexes purified from WT, *tra1-Sptra2*, and *tra1-mTOR* strains, using Spt7 as the bait. Numbers at the bottom of the gel represent LFQ intensity ratios of Tra1 to Spt7, from LC-MS/MS analyses of purified SAGA complexes.
Values for each mutant are expressed as percentage of WT SAGA (ND: not detected).
Shown are gels that are representative of two independent experiments.

b LC-MS/MS analysis of TTT complexes purified from WT, *tra1-Sptra2*, and *tra1-Sctra1* strains (see Fig. 5b), using Tti2 as the bait. LFQ intensity ratios of Tra1 to Tti2 from 1-3 biological replicates are plotted individually with the mean (black bar).

Source data are provided as a Source Data file.

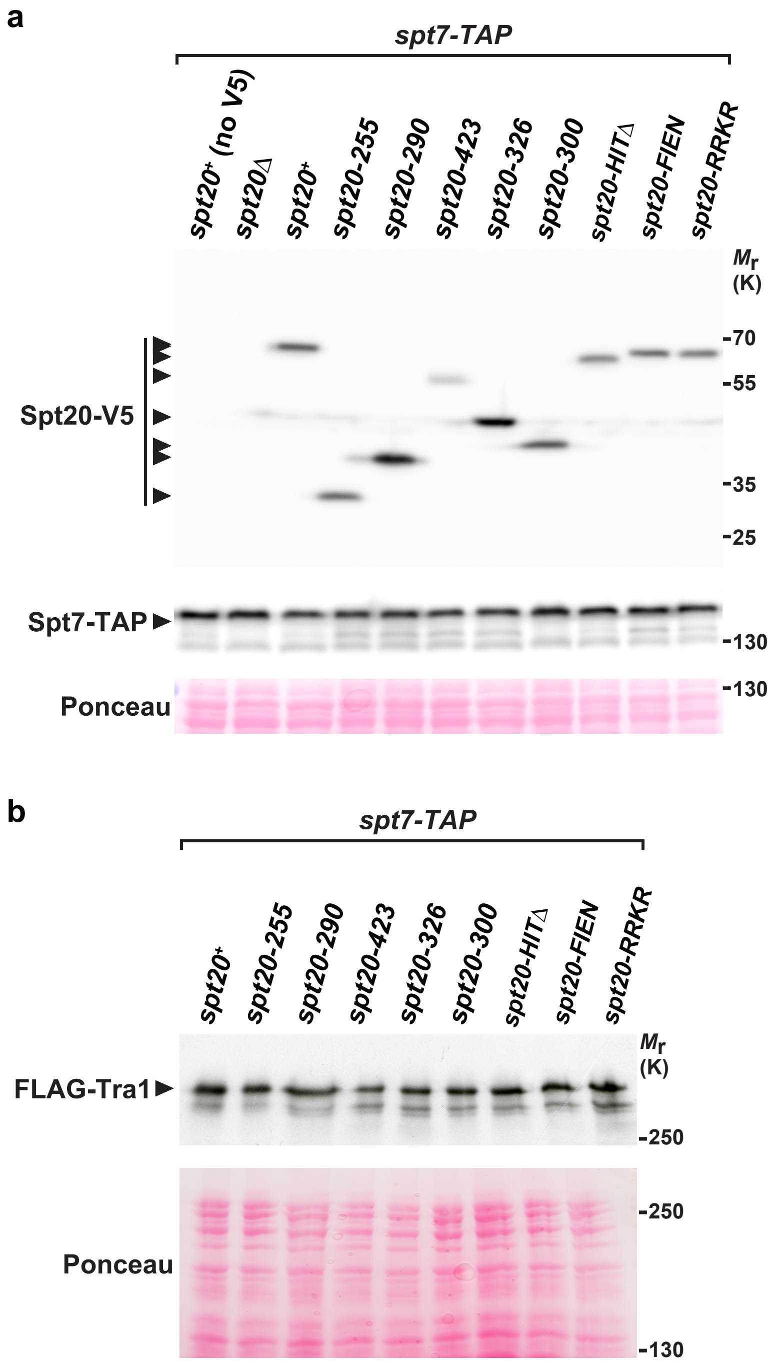

Supplementary Figure 10. Expression levels of Tra1, Spt7, and Spt20 in truncation and point mutants.

a Anti-V5 and anti-HA Western blot analyses of Spt20-V5 (upper blot) and Spt7-TAP (lower blot) in a fraction of the input used for TAPs shown in Figure 5. Ponceau red staining is used as loading control.

b Anti-FLAG Western blot analyses of FLAG-Tra1 in total extracts from all *spt20* mutants analyzed in Figure 5. Ponceau red staining is used as loading control.

Source data are provided as a Source Data file.

Supplementary Table 1. Quantification of SAGA subunit levels in Spt7 affinity purifications from *spt20* mutants. Shown are LFQ intensities from LC-MS/MS analyses of total protein mixtures from purification eluates in two distinct experiments, analyzing either a wild-type and an *spt20-255* N-terminal truncation mutant, or a wild-type and an *spt20*Δ deletion mutant.

Protein name	LFQ intensity [WT]	LFQ intensity [*spt20-255*]	LFQ intensity [WT]	LFQ intensity [*spt20*Δ]
Tra1	1.304E+10	7.565E+05	2.862E+11	1.080E+09
Spt7 (bait)	2.566E+09	5.431E+09	2.793E+11	4.079E+11
Taf5	5.140E+09	8.261E+09	1.982E+11	2.413E+11
Taf12	3.304E+09	4.129E+09	8.216E+10	7.383E+10
Spt8	2.508E+09	3.589E+09	1.258E+11	1.551E+11
Ada3	3.639E+09	4.227E+09	1.617E+11	1.372E+11
Taf6	8.444E+09	8.392E+09	1.475E+11	2.140E+11
Gcn5	3.218E+09	4.727E+09	9.968E+10	1.041E+11
Spt20	8.949E+09	ND	1.196E+11	ND
Ada2	4.779E+09	3.994E+09	1.148E+11	1.010E+11
Ada1	1.944E+09	9.970E+08	1.058E+11	1.089E+11
Sgf29	4.713E+09	4.146E+09	6.270E+10	6.295E+10
Spt3	2.287E+09	1.762E+09	5.919E+10	3.321E+10
Taf9	1.579E+09	3.128E+09	4.791E+10	5.324E+10
Taf10	2.443E+09	3.543E+09	4.698E+10	5.502E+10
Sgf73	4.904E+08	ND	4.259E+09	ND
Ubp8	3.803E+08	ND	8.479E+09	ND
Sus1	4.536E+08	ND	1.832E+09	ND
Sgf11	1.064E+08	ND	7.554E+08	ND

ND: no MS/MS count detected

Supplementary Table 2. Quantification of Tra1, Sgf73, and Ubp8 levels in Spt7 affinity purifications from *spt20* and *tra1* mutants. Shown are relative LFQ intensity ratios of each subunit, normalized to the bait, Spt7, from LC-MS/MS analyses of total protein mixtures from purification eluates. Spt7 was purified from wild-type (WT), spt20-290, spt20-300, spt20-HITD, spt20-FIEN, spt20-RRKR, tra1-Sptra2, and tra1-Sctra1 strains. Values from the WT sample were set at to 1, allowing to allow comparisons across mutants.

	**LFQ ratio [WT]**	**LFQ ratio [*spt20-290*]**	**LFQ ratio [*spt20-300*]**	**LFQ ratio [*spt20-HIT* Δ]**	**LFQ ratio [*spt20-FIEN*]**	**LFQ ratio [*spt20-RRKR*]**
Tra1/Spt7	1.000	0.003	1.067	0.026	0.020	0.509
Sgf73/Spt7	1.000	0.410	2.098	0.268	0.166	0.568
Ubp8/Spt7	1.000	0.372	1.991	0.430	0.115	0.585

	**LFQ ratio [WT]**	**LFQ ratio [*tra1-Sptra2*]**	**LFQ ratio [*tra1-Sctra1*]**
Tra1/Spt7	1.000	0.000	1.138
Sgf73/Spt7	1.000	0.173	1.158
Ubp8/Spt7	1.000	0.215	1.266

Supplementary Table 3. Quantification of SAGA subunit levels in Ada1 affinity purifications from *sgf73* mutants. Shown are LFQ intensities from LC-MS/MS analyses of total protein mixtures from purification eluates in one wild-type and two independent *sgf73* Δ mutant strains.

Protein name	LFQ intensity [WT]	LFQ intensity [*sgf73* Δ]	LFQ intensity [*sgf73* Δ]
Tra1	7.259E+10	7.294E+10	3.454E+10
Ada1 (bait)	2.333E+10	3.588E+10	2.609E+10
Spt7	1.229E+11	1.354E+11	1.636E+11
Taf5	6.928E+10	8.487E+10	9.101E+10
Ada3	4.012E+10	4.992E+10	6.311E+10
Taf12	1.938E+10	2.424E+10	3.459E+10
Spt8	2.813E+10	3.425E+10	4.102E+10
Gcn5	4.455E+10	5.227E+10	6.056E+10
Spt20	4.210E+10	5.506E+10	6.421E+10
Taf6	2.205E+10	4.287E+10	3.006E+10
Ada2	1.271E+10	2.685E+10	1.506E+10
Sgf29	1.439E+10	2.045E+10	1.838E+10
Spt3	9.019E+09	1.753E+10	1.142E+10
Taf9	1.461E+10	2.215E+10	2.364E+10
Taf10	8.445E+09	1.165E+10	1.374E+10
Ubp8	1.292E+09	ND	ND
Sgf73	1.774E+09	ND	ND
Sgf11	3.435E+08	ND	ND
Sus1	5.391E+08	ND	ND

ND: no MS/MS count detected

Supplementary Table 4. List of *S. pombe* and *S. cerevisiae* strains used in this study.

Strain	Genotype		Source
DHP42	*h+*		Lab stock
DHP43	*h-*		Lab stock
DHP1139	*h-*	*tti2-GLY6-HA3-TAP2::kanMX6*	This study
DHP609	*h-*	*tra1Δ::kanMX6*	This study
DHP1235	*h-*	*leu1-32 ars1::pRad15-Cre-EBD-LEU2*	Derived from A9823 (R. Allshire)
DHP1487	*h-*	*leu1-32 ars1::pRad15-Cre-EBD-LEU2 lox::kanMX6::tti2-lox-HA*	This study
DHP1488	*h-*	*leu1-32 ars1::pRad15-Cre-EBD-LEU2 lox::kanMX6::tra2::lox*	This study
DHP1489	*h-*	*leu1-32 ars1::pRad15-Cre-EBD-LEU2 lox::kanMX6::tti2-lox-HA spt7-MYC13::natMX6*	This study
DHP1490	*h-*	*leu1-32 ars1::pRad15-Cre-EBD-LEU2 lox::kanMX6::tti2-lox-HA epl1-MYC13::natMX6*	This study
DHP782	*h-*	*spt7-HA3-TAP2::kanMX6*	Lab stock
DHP1293	*h-*	*epl1-HA3-TAP2::kanMX6*	This study
DHP1295	*h-*	*mst1-HA3-TAP2::kanMX6*	This study
DHP1492	*h-*	*tra1Δ::hphMX6 spt7-HA3-TAP2:kanMX6*	This study
DHP1491	*h-*	*leu1-32 ars1::pRad15-Cre-EBD-LEU2 lox::kanMX6::tti2-lox-HA spt7-HA3-TAP2::kanMX6*	This study
DHP1493	*h-*	*leu1-32 ars1::pRad15-Cre-EBD-LEU2 lox::kanMX6::tti2-lox-HA mst1-HA3-TAP2::kanMX6*	This study
DHP1494	*h-*	*leu1-32 ars1::pRad15-Cre-EBD-LEU2 lox::kanMX6::tra2::lox epl1-HA3-TAP2::kanMX6*	This study
DHP1495	*h-*	*leu1-32 ars1::pRad15-Cre-EBD-LEU2 lox::kanMX6::tra2::lox vid21-TAP2::natMX6*	This study
DHP1549	*h-*	*leu1-32 ars1::pRad15-Cre-EBD-LEU2 lox::kanMX6::tra2::lox spt7-HA3-TAP2::kanMX6*	This study
DHP1188	*h-*	*ade6::ade6-Padh15-skp1-OsTIR1-natMX6-Padh15-skp1-AtTIR1-2NLS*	Derived from HM2985 (H. Masukata)
DHP1179	*h-*	*ade6::ade6-Padh15-skp1-OsTIR1-natMX6-Padh15-skp1-AtTIR1-2NLS tel2-HA3-AID3::kanMX6*	This study
DHP1496	*h-*	*leu1-32 ars1::pRad15-Cre-EBD-LEU2 natMX6::RI-tra1 spt7-HA3-TAP2::kanMX6 ade6::ade6-Padh15-skp1-OsTIR1-natMX6-Padh15-skp1-AtTIR1-2NLS tel2-HA3-mAID3::kanMX6*	This study
DHP1497	*h+*	*leu1-32 ars1::pRad15-Cre-EBD-LEU2 natMX6::RI-tra1 spt7-HA3-TAP2::kanMX6 hsp90-201::kanMX6*	This study
DHP1498	*h-*	*leu1-32 ars1::pRad15-Cre-EBD-LEU2 natMX6::RI-tra1 spt7-HA3-TAP2::kanMX6*	This study
DHP852	*h-*	*ura4::fbp1-lacZ hsp90-201::kanMX6*	Derived from CHP879 (C. Hoffman)
DHP1521	*h+*	*hsp90-26*	Derived from RA1193 (P. Russell)
DHP1522	*h+*	*hsp90-26 spt7-HA3-TAP2::kanMX6*	This study
DHP1432	*h-*	*tra1-Sptra2*	This study
DHP1425	*h-*	*tra1-Sctra1*	This study
DHP1483	*h-*	*FLAG3-tra1-Sptra2*	This study
DHP1532	*h-*	*FLAG3-tra1-Sctra1*	This study
DHP1523	*h-*	*FLAG3-tra1 spt7-HA3-TAP2::kanMX6*	This study
DHP1514	*h-*	*FLAG3-tra1-Sptra2 spt7-HA3-TAP2::kanMX6*	This study
DHP1515	*h-*	*FLAG3-tra1-Sctra1 spt7-HA3-TAP2::kanMX6*	This study
DHP1373	*h+*	*ura4-D18 leu1-32 ade6-M216 spt7-HA3-TAP2::kanMX6 tra1-mTOR*	This study
DHP1434	*h-*	*tra1-Sptra2 tti2-GLY6-HA3-TAP2::kanMX6*	This study

DHP1424	*h-*	*tra1-Sctra1 tti2-GLY6-HA3-TAP2::kanMX6*	This study
DHP1318	*h-*	*spt20Δ::natMX6 spt7-HA3-TAP2::kanMX6*	This study
DHP1519	*h-*	*spt20-PK3::hphMX6*	This study
DHP1358	*h-*	*spt20-255-PK3::hphMX6 spt7-HA3-TAP2::kanMX6*	This study
DHP1365	*h-*	*spt20-300-PK3::hphMX6 spt7-HA3-TAP2::kanMX6*	This study
DHP1380	*h-*	*spt20-290-PK3::hphMX6 spt7-HA3-TAP2::kanMX6*	This study
DHP1382	*h-*	*spt20-423-PK3::hphMX6 spt7-HA3-TAP2::kanMX6*	This study
DHP1401	*h-*	*spt20-326-PK3::hphMX6 spt7-HA3-TAP2::kanMX6*	This study
DHP1409	*h-*	*ura4-D18 ade6-M216 spt20-PK3::hphMX6 spt7-HA3-TAP2::kanMX6*	This study
DHP1421	*h-*	*ura4-D18 ade6-M216 spt20-HITΔ-PK3::hphMX6 spt7-HA3-TAP2::kanMX6*	This study
DHP1444	*h-*	*ura4-D18 ade6-M216 spt20-RRKR:AAAA-PK3::hphMX6 spt7-HA3-TAP2::kanMX6*	This study
DHP1446	*h-*	*ura4-D18 ade6-M216 spt20-FIEN:AAAA-PK3::hphMX6 spt7-HA3-TAP2::kanMX6*	This study
DHP1566	*h-*	*FLAG3-tra1 sgf11-MYC13::natMX6 spt7-HA3-TAP2::kanMX6 spt20D255aa-PK3::hphMX6*	This study
DHP1565	*h-*	*FLAG3-tra1 sgf11-MYC13::natMX6 spt20D290aa-PK3::hphMX6*	This study
DHP1547	*h+*	*FLAG3-tra1 spt7-HA3-TAP2::kanMX6 spt20D423aa-PK3::hphMX6*	This study
DHP1543	*h-*	*FLAG3-tra1 spt7-HA3-TAP2::kanMX6 spt20D327aa-PK3::hphMX6*	This study
DHP1564	*h-*	*FLAG3-tra1 sgf11-MYC13::natMX6 spt7-HA3-TAP2::kanMX6 spt20D301aa-PK3::HphMX6*	This study
DHP1562	*h-*	*FLAG3-tra1 sgf11-HA3-TAP2::kanMX6 spt20-HITΔ-PK3::hphMX6*	This study
DHP1563	*h-*	*FLAG3-tra1 sgf11-HA3-TAP2::kanMX6 spt20-FIEN:AAAA-PK3::hphMX6*	This study
DHP1553	*h-*	*FLAG3-tra1 spt7-HA3-TAP2::kanMX6 spt20-RRKR:AAAA-PK3::hphMX6*	This study
DHP1516	*h-*	*spt20Δ::kanMX6*	This study
DHP1517	*h-*	*spt20-HITΔ-PK3::hphMX6*	This study
DHP1448	*MATa*	*ura3D0 leu2D1 trp1D63 hisD4-917 lys2-173R2 HA-SPT7-TAP::TRP1*	FY2031 (F. Winston)
DHP1460	*MATa*	*ura3D0 leu2D1 trp1D63 hisD4-917 lys2-173R2 HA-SPT7-TAP::TRP1 spt20-D492aa-PK3::hphMX6*	This study
DHP1462	*MATa*	*ura3D0 leu2D1 trp1D63 hisD4-917 lys2-173R2 HA-SPT7-TAP::TRP1 spt20-D380aa-PK3::hphMX6*	This study
DHP1524	*MATa*	*ura3D0 leu2D1 trp1D63 hisD4-917 lys2-173R2 HA-SPT7-TAP::TRP1 spt20-D474aa-PK3::hphMX6*	This study
DHP1538	*MATa*	*ura3D0 leu2D1 trp1D63 hisD4-917 lys2-173R2 HA-SPT7-TAP::TRP1 spt20-D408aa-PK3::kanMX6*	This study
DHP1520	*h-*	*ura4-D18 tra1Δ::ura4 spt7-HA3-TAP2::kanMX6 sgf11-MYC13::natMX6*	This study
DHP1499	*h-*	*leu1-32 ars1::pRad15-Cre-EBD-LEU2 natMX6::RI-tra1 spt7-HA3-TAP2::kanMX6 sgf11-MYC13::natMX6*	This study
DHP2	*h-*	*ura4-D18 ade6-M210 ada1-HA3-TAP2::kanMX6*	Lab stock
DHP486	*h-*	*ura4-D18 ade6-M216 sgf73Δ::ura4 ada1-HA3-TAP2::kanMX6*	This study

Supplementary Table 5. List of oligonucleotides used in this study.

Gene	Name	Description[a]	Coordinates[b]	Sequence
	DHO 1176	Subcloning HA tag (DHB137)		AGCTGAAGCTTCGTACGCTGCAGGTACCCATACGATGTTCCTGACTATGC
	DHO 1177	Subcloning HA tag (DHB137)		GCACCAGCACCAGCACCGGCACCGGCAGCGTAATCTGGAACGTCATATGG
	DHO 1513	Subcloning V5-PK tag (DHB123)		TCGATGATCCCAAACCCATTGTTGGGTTTGGACGGT
	DHO 1514	Subcloning V5-PK tag (DHB123)		TCGAACCGTCCAAACCCAACAATGGGTTTGGGATCA
act1	DHO 90	RT-qPCR	+10 to +29	GAAATCGCAGCGTTGGTTAT
act1	DHO 91	RT-qPCR	+186 to +167	ACGCTTGCTTTGAGCTTCAT
epl1	DHO 1695	C-terminal tagging / pFA6a		CAATTATACTCGATGATGAATCTGATGCTTCTATAATGTTTCGAGCTAGCTTGCTAAACGATGATATGGGTACTCAGTCACGGATCCCCGGGTTAATTAA
epl1	DHO 1696	C-terminal tagging / pFA6a		CGAGGAGAAATAGTTAAAATAAAATTTGATCGAAATCTTTAGTAATGGAAATATTATAAATACCATAGATAAAAAGCGTTGAATTCGAGCTCGTTTAAAC
gst2	DHO 1900	RT-qPCR	+233 to +252	TGGCTGACAAGTATGATACG
gst2	DHO 1901	RT-qPCR	+400 to +382	TGACTGCAGATACCACTGG
mei2	DHO 188	ChIP-qPCR	-1946 to -1924	GAGCTTACTGACATCGCATCA
mei2	DHO 189	ChIP-qPCR	-1721 to -1701	TCAACAGCAGCTTTCAGTGG
mst1	DHO 1699	C-terminal tagging / pFA6a		AAAGAAGAAGGATCAATGGAGATTTGTTGGCAGATTGGCAACCTCCTGTTTTTCACCCTTCCCAACTACGATTTGGATGGCGGATCCCCGGGTTAATTAA
mst1	DHO 1700	C-terminal tagging / pFA6a		ACATGTGGGCTAGTATACGATATCCAGCTATCCTGCAAAATGGAGCAGCCGAAAAAACCAAGGCAATAGCAGAGGCTTTTGAATTCGAGCTCGTTTAAAC
pho84	DHO 1300	ChIP-qPCR	-477 to -458	CCGCGTTACCTTAATCCTGA
pho84	DHO 1301	ChIP-qPCR	-250 to -269	AGCTTTGGTGTTGACGTTCC
sgf11	DHO 1715	C-terminal tagging / pFA6a		AGAAATGTAAAGGCAGGTCACTTTCAAACGCTACCTCGTACTCAACTCTTTTCGAAGATGACAATGCAGACGAAGAGGACCGGATCCCCGGGTTAATTAA
sgf11	DHO 1716	C-terminal tagging / pFA6a		ATAATATTGTAGTTCGTCTTACTGGGAAAGACATACATGTGCATAATGAGAATAAACCTTAAAACAAGCAACAAATAAAAGAATTCGAGCTCGTTTAAAC
SPAC977.12	DHO 1890	RT-qPCR	+123 to +142	TGCAATGGGTGGTACAATCG
SPAC977.12	DHO 1891	RT-qPCR	+311 to +292	AGTTTTAGGACATCAGCAGG
SPCC1884.01	DHO 1892	RT-qPCR	+929 to +949	AGAACATCATCGTTCGCTTGG
SPCC1884.01	DHO 1893	RT-qPCR	+1111 to +1093	TTGGATATCGACCTCTTTGG
SPCC569.05c	DHO 1896	RT-qPCR	+124 to +143	TCACAGCTTTCTCATTCTGC
SPCC569.05c	DHO 1897	RT-qPCR	+343 to +324	AGACAAACCGATCAGGATCC
spt20	DHO 849	Deletion / pFA6a		TTAACCAGCTTATAATTTTAATTGTAAACATAGGGGGAATATTGTTTTACAATTACTGGTATAGTTCGTTGAACTAAGAGCGGATCCCCGGGTTAATTAA
spt20	DHO 850	Deletion & C-terminal tagging / pFA6a		TATGCAACAAAAAAAATAAAATTTTAAAAATAATAAATAAAAAACTAAATTCGTGTATTAAATGATGAAGCATTTGAGAGGAATTCGAGCTCGTTTAAAC
spt20	DHO 1592	C-terminal tagging / pFA6a		TGACGGTTGCACGAAATCTTCATTTTCAACAACTCCAAATGAGACAGCAGCAACAACAGCAGCAAATGAAGGCTGAAAGGCGGATCCCCGGGTTAATTAA
spt20	DHO 1558	C-terminal deletion (256-474) / pFA6a	+764 to +843	AGTCTAAAGCGGAAATGATTCAATTTATGAACCAGTTGGCCGACTCTGCACCACCATGCACCAGAAAGAAACCTCAAGGACGGATCCCCGGGTTAATTAA
spt20	DHO 1559	C-terminal deletion (256-474) / pFA6a	+923 to +844	CGTTTTCTTTGGTCATCCATTAGTAACAAAAGGTTCTCTTTTTCAAGTCTTTCTGCTTCTTCATCAGCAAGTTGGGCTGAGAATTCGAGCTCGTTTAAAC
spt20	DHO 1560	C-terminal deletion (291-474) / pFA6a	+769 to +948	CAGAAAGACTTGAAAAAGAGAACCTTTTGTTACTAATGGATGACCAAAGAAAACGCGATTTTCAACCTACCTTTCAAAGACGGATCCCCGGGTTAATTAA
spt20	DHO 1561	C-terminal deletion (291-474) / pFA6a	+1028 to +949	GCTTTTTGTTGCTGTTGCATTTGCATTTGCCTTTGTTGTAGAATAGCTCGTTTACGCCGCACATTCTCAATAAATTGTAGGAATTCGAGCTCGTTTAAAC
spt20	DHO 1650	C-terminal deletion (301-474) / pFA6a	+902 to +981	TAATGGATGACCAAAGAAAACGCGATTTTCAACCTACCTTTCAAAGACTACAATTTATTGAGAATGTGCGGCGTAAACGACGGATCCCCGGGTTAATTAA
spt20	DHO 1651	C-terminal deletion (301-474) / pFA6a	+1061 to +982	GGTTGCTGAGCTTTCGGTGATTGCTGCTGCTGCGCTTTTTGTTGCTGTTGCATTTGCATTTGCCTTTGTTGTAGAATAGCGAATTCGAGCTCGTTTAAAC
spt20	DHO 1652	C-terminal deletion (327-474) / pFA6a	+980 to +1059	GAGCTATTCTACAACAAAGGCAAATGCAAATGCAACAGCAACAAAAAGCGCAGCAGCAGCAATCACCGAAAGCTCAGCAACGGATCCCCGGGTTAATTAA
spt20	DHO 1653	C-terminal deletion (327-474) / pFA6a	+1139 to +1060	ATACTACTAGGTGGTAGACGTTGGATTTTGGGTGTAGTTTTTCTTTGAACTGGAGCAGACTGAACCAAATGTGCGGGTGGAATTCGAGCTCGTTTAAAC
spt20	DHO 1562	C-terminal deletion (424-474) / pFA6a	+1355 to +1434	GAGAATCCGTGGATTTGATAAAAATACGACAACTTGCAATTTTGTTTCAACAGCGTGCCTCTCAATTAAAAGCTAGAGGTCGGATCCCCGGGTTAATTAA
spt20	DHO 1563	C-terminal deletion (424-474) / pFA6a	+1514 to +1435	ACCGTCATTACTGTTGCCAAATCAGTACCAGCGGCTATTGCTTGACGATTCAGAATTTCAGTAATTTGTTCCCGTGTAGCGAATTCGAGCTCGTTTAAAC
spt20	DHO 1485	N-terminal deletion (1-241) / nmt-HA	+805 to +884	TTTTCAAGTCTTTCTGCTTCTTCATCAGCAAGTTGGGCTGATCCTTGAGGTTTCTTTCTGGTGCATGGTGGTGCAGAGTCGCACTGAGCAGCGTAATCTG
spt20	DHO 1486	N-terminal deletion (1-241) / pFA6a	-80 to -1	TTAACCAGCTTATAATTTTAATTGTAAACATAGGGGGAATATTGTTTTACAATTACTGGTATAGTTCGTTGAACTAAGAGGAATTCGAGCTCGTTTAAAC
spt20	DHO 1672	Internal deletion (290-300) / pKS-ura4	+769 to +948	CAGAAAGACTTGAAAAAGAGAACCTTTTGTTACTAATGGATGACCAAAGAAAACGCGATTTTCAACCTACCTTTCAAAGACGCCAGGGTTTTCCCAGTCACGAC
spt20	DHO 1673	Internal deletion (290-300) / pKS-ura4	+1061 to + 982	GGTTGCTGAGCTTTCGGTGATTGCTGCTGCTGCGCTTTTTGTTGCTGTTGCATTTGCATTTGCCTTTGTTGTAGAATAGCAGCGGATAACAATTTCACACAGGA
spt20	DHO 1660	RIP-qPCR	+1425 to +1444	AAGCTAGAGGTGCTACACGG
spt20	DHO 1661	RIP-qPCR	+1563 to +1543	TGTTGCTGCTGTCTCATTTGG
	DHO 1759	Subcloning HIT-BamHI (DHB179 & DHB193)		ATCGGATCTGGTTCCGCGTG
	DHO 1760	Subcloning HIT-XhoI (DHB179 & DHB193)		CAGTCACGATGCGGCCGCT
SPT20	DHO 1845	C-terminal tagging / pFA6a		GTACTTTAATACAATATATATATATATATATATATATATATATATATATATATAAGGAATGATAACTCTATTTAAGTAGAGAATTCGAGCTCGTTTAAAC
SPT20	DHO 1847	C-terminal deletion (380-604) / pFA6a	+1061 to +1140	AAAAATCCTTCATTCACGAACATCGTGCTGAGTCTACGAGAGAAGGCACGAAGGGCGTTGTAGGGCATATCGAAGAACGCCGGATCCCCGGGTTAATTAA
SPT20	DHO 1965	C-terminal deletion (408-604) / pFA6a	+1108 to +1187	TGATGTTGATCATGAATGAACGCACAACAACGATAACCAACTCCACGTTTGCTGTTTCGTTAACCAAGAATGCGATGGAGCGGATCCCCGGGTTAATTAA
SPT20	DHO 1849	C-terminal deletion (492-604) / pFA6a	+1397 to 1476	CTACCATGGGTAACGATAATAATCAATTTAGCAGGCTGAAATTTATTGAACAATGGAGAATAAATAAAGAAAAAAGAAAACGGATCCCCGGGTTAATTAA
SPT20	DHO 1964	C-terminal deletion (474-604) / pFA6a	+1343 to + 1422	CTAATGGCAATCAAGTAGCTTTGGCAGCGGCAGCGGCTGCGGCTGCAGTGGGATCTACCATGGGTAACGATAATAATCAACGGATCCCCGGGTTAATTAA
ssa2	DHO 1242	ChIP-qPCR	-429 to -410	CACGCTCTTCCAGGACTTTC
ssa2	DHO 1243	ChIP-qPCR	-248 to -267	TCTCGAAGTTTCCCAAAACC
tra1	DHO 1424	Internal deletion (2624-2674) / pKS-ura4	+8022 to +8101	GATTCACGATTATTAGACCAATCGCTAATTCTCCAAGCACACTCGAGCAATAGTTCAGAAGATCCCTCGTGCTTCGCCAAAGCGGATAACAATTTCACACAGGA
tra1	DHO 1425	Internal deletion (2624-2674) / pKS-ura4	+7790 to +7869	AAGATGATATGTTTTATGGCCATTGGCGACGAAACTGTAAGTACTTGCAAACTCAAGTTGCTCTCTCGTATGAGCAACTTCGCCAGGGTTTTCCCAGTCACGAC
tra1	DHO 1764	CSI sgRNA-1	+7935 to +7916	ATAGTTGCTGTTGCCAAAAAACATAACCTGTACCGAAGAAAATGGCTTCACTGCGAGCCTgttttagagctagaaatagcaag
tra1	DHO 1763	CSI sgRNA-2	+7862 to +7881	ATAGTTGCTGTTGCCAAAAAACATAACCTGTACCGAAGAAAGCAACTTGGAATGTGGGGAgttttagagctagaaatagcaag
tra1	DHO 1819	ATG sgRNA-1	+96 to +115	ATAGTTGCTGTTGCCAAAAAACATAACCTGTACCGAAGAAGCAGAGATCCAAGCATCTGAgttttagagctagaaatagcaag
tra1	DHO 1820	ATG sgRNA-2	+126 to +107	ATAGTTGCTGTTGCCAAAAAACATAACCTGTACCGAAGAATGATACATCCATCAGATGCTgttttagagctagaaatagcaag
tra2	DHO 1593	3' loxP insertion / pUG6	+10950 to +11029	CTTTGGTGGATTTAGTATCTCAAGCAACTAATCCTCAGCAGCTGGCGCAAATGGATCAACTTTGGCAAGCTTGGCTTTAAAGCTGAAGCTTCGTACGCTGC
tra2	DHO 1594	3' loxP insertion / pUG6	+11110 to +11030	ATTTTCTCCTAATAATAAATAATCTGAGCATTCGTAGCAATTCAATATTAGATGAAACCTCAAAACTGATTTGCAATTTGTATAGGGAGACCGGCAGATCC
tra2	DHO 1615	5' loxP insertion / pFA6a	-231 to -166	TTGTATTTGATAAAAATTACACTCACGTCGATTTCAAATAGCTTCAGTGGCTTCTTGAATAGTGCAATAACTTCGTATAATGTATGCTATACGAAGTTATCGGATCCCCGGGTTAATTAA
tra2	DHO 1616	5' loxP insertion / pFA6a	-86 to -165	AAAAGCACAGAACGATTACTGTCAGATGTTGTTTAGTTGTTGCTATGTTCAAGGTGAATCGTATAGTGAGGACGCAAAACGAATTCGAGCTCGTTTAAAC
tra2	DHO 388	RT-qPCR	+9307 to +9326	CTCGTTTTCAAGCGTTAGCC
tra2	DHO 389	RT-qPCR	+9547 to +9528	AAGAGCGACAATGAGCCTGT

trk2	DHO 1711	RT-qPCR	+1642 to +1661	TCTTGGTTTGGTTGCGTTCC
trk2	DHO 1712	RT-qPCR	+1789 to +1770	ATCGCTGTTCAACGAATAGC
tti2	DHO 819	C-terminal tagging / pFA6a		TAAAATTGAACATAACTGTTGAGCAACTCGGTGATTACAATGTAATATTGCCACTGCTTGAACCCCTGCAAATCCCACTTGGGGGAGGCGGGGGTGGATACCCATACGATGTTCCTGA
tti2	DHO 1222	C-terminal tagging / pFA6a		TAAAATTGAACATAACTGTTGAGCAACTCGGTGATTACAATGTAATATTGCCACTGCTTGAACCCCTGCAAATCCCACTTGGGGGAGGCGGGGGTGGA
tti2	DHO 820	Deletion & C-terminal tagging / pFA6a		TTTTTCGAATAACCATTGTTACCAATTTATCTAGGTTGAAGGTTGGTTAGTAATGAGGAGAGTCTTTCCTTATATGTACCGAATTCGAGCTCGTTTAAAC
tti2	DHO 1522	3' loxP insertion / pUG75	+1576 to +1497	TTTTTCGAATAACCATTGTTACCAATTTATCTAGGTTGAAGGTTGGTTAGTAATGAGGAGAGTCTTTCCTTATATGTACCAGGGAACAAAAGCTTGCATG
tti2	DHO 1523	3' loxP insertion / pUG75	+1414 to +1493	TAAAATTGAACATAACTGTTGAGCAACTCGGTGATTACAATGTAATATTGCCACTGCTTGAACCCCTGCAAATCCCACTTGGTGGATCTGGTGGATCT
tti2	DHO 1524	5' loxP insertion / pUG6	-387 to -308	CTTGGTAATAACTATAAAAAACAATAATGCTTATATACTCAATAGAACTTATATAGCTAAAGCGATAAGGTAAAGTACCTGGTCGACAACCCTTAATATA
tti2	DHO 1525	5' loxP insertion / pUG6	-228 to -307	AATTTAATAAATTAACAATTTACTAGTTTTTAAAGGTATAAAAAGCAAAATTTTTAGTAACTAACATCAATTCATTACACATTAAGGGTTCTCGAGAGCT
vid21	DHO 1821	C-terminal tagging / pFA6a		GTTCTTTAAAACTAACACCTGAACAAATTCATCAGTTGCAGCAAAGGAAGCAAACTGTACCTACTACTGAAAGGACACAGCGGATCCCCGGGTTAATTAA
vid21	DHO 1822	Deletion & C-terminal tagging / pFA6a		CTCGCCTCGTAATTTCCTTCTAATATACAACAGCACTCAAAAAAAATACAATAAATCGAAAAATTGCATTGAGGCGATACGAATTCGAGCTCGTTTAAAC

[a] pFA6a, pKSura4 and DHB plasmids are defined in Materials and Methods

[b] Coordinates are relative to the ATG of each ORF (A defined as +1)

Supplementary Table 6. Cross referencing between MaxQuant results and raw data files available on ProteomeXchange (dataset ID: PXD013256).

Supplementary Data 1

- SAGA_150911_E_43.raw
- SAGA_150911_E_99.raw
- SAGA_150911_E_115.raw
- SAGA_150911_E_184.raw
- MaxQuant (v1.5.0.0), Database: Proteome_spombe-iso_2015_04.fasta

- SAGA_170310_E_43.raw
- SAGA_170310_E_612.raw
- SAGA_170310_E_623A.raw
- SAGA_170310_E_623B.raw
- SAGA_170310_E_955A.raw
- SAGA_170310_E_955C.raw
- SAGA_170310_E_955D.raw
- SAGA_170310_E_962.raw
- MaxQuant (v1.5.5.1), Databases: RefProteome_SPOMBE-cano_2017_01.fasta TOR1_SCHPO_Mutant.fasta / TRA1_SCHPO_Mutant.fasta

- SAGA_171116_TAP11_P_1.raw
- SAGA_171116_TAP11_P_2.raw
- SAGA_171116_TAP11_P_3.raw
- SAGA_171116_TAP11_P_4.raw
- SAGA_171116_TAP11_P_5.raw
- SAGA_171116_TAP11_P_6.raw
- MaxQuant (v1.5.5.1), Database: RefProteome_SPOMBE-cano_2017_10.fasta

- SAGA_180410_TAP13_P_01.raw
- SAGA_180410_TAP13_P_02.raw
- SAGA_180410_TAP13_P_03.raw
- SAGA_180410_TAP13_P_04.raw
- SAGA_180410_TAP13_P_05.raw
- SAGA_180410_TAP13_P_06.raw
- MaxQuant (v1.5.5.1), Database: RefProteome_SPOMBE-cano_2017_10.fasta

Figure 2a,b

- SAGA_180327_DHP1151b_P_31.raw
- SAGA_180327_DHP1151b_P_31B.raw
- SAGA_180327_DHP1151_P_31.raw
- SAGA_180327_DHP1151_P_31B.raw
- SAGA_180327_DHP1154-1_P_31B.raw
- SAGA_180327_DHP1154-1b_P_31B.raw
- SAGA_180327_DHP1154-2_P_31B.raw
- SAGA_180327_DHP1154-2b_P_31B.raw
- MaxQuant (v1.5.5.1), Database: RefProteome_SPOMBE-cano_2017_10.fasta

Figure 3d

- SAGA_180816_TAP07_P_A.raw
- SAGA_180816_TAP07_P_B.raw
- SAGA_180816_TAP07_P_C.raw
- SAGA_180816_TAP07_P_D.raw
- MaxQuant (v1.5.5.1), Database: RefProteome_SPOMBE-cano_2017_10.fasta

- SAGA_180816_TAP08_P_A.raw
- SAGA_180816_TAP08_P_B.raw
- SAGA_180816_TAP08_P_C.raw
- SAGA_180816_TAP08_P_D.raw
- MaxQuant (v1.5.5.1), Database: RefProteome_SPOMBE-cano_2017_10.fasta

- SAGA_180816_TAP09_P_DMSO_A.raw
- SAGA_180816_TAP09_P_DMSO_E.raw
- SAGA_180816_TAP09_P_DMSO-C.raw
- SAGA_180816_TAP09_P_BE_B.raw
- SAGA_180816_TAP09_P_BE_D.raw
- SAGA_180816_TAP09_P_BE_F.raw
- MaxQuant (v1.5.5.1), Database: RefProteome_SPOMBE-cano_2017_10.fasta

Figure 4b

- SAGA_180725_TAP15_P_21KO.raw
- SAGA_180725_TAP15_P_21WT.raw
- SAGA_180725_TAP15_P_24KO.raw
- SAGA_180725_TAP15_P_24WT.raw
- MaxQuant (v1.5.5.1), Database: RefProteome_SPOMBE-cano_2017_10.fasta

Figure 5c

- SAGA_181120_TAP44_P_782.raw
- SAGA_181120_TAP44_P_1435.raw
- SAGA_181120_TAP44_P_1441.raw
- MaxQuant (v1.5.5.1), Database: RefProteome_SPOMBE-cano_2017_10.fasta

Figure 6a,c,e

- SAGA_170428_DHP_P_782.raw
- SAGA_170428_DHP_P_1318.raw
- SAGA_170428_DHP_P_1329.raw
- SAGA_170428_DHP_P_1336.raw
- MaxQuant (v1.5.5.1), Database: RefProteome_SPOMBE-cano_2017_01.fasta

- SAGA_180327_TAP30_P_1365.raw
- SAGA_180327_TAP30_P_1380.raw
- SAGA_180327_TAP30_P_dTID4.raw
- SAGA_180327_TAP30_P_1444.raw
- SAGA_180327_TAP30_P_1446.raw
- SAGA_180327_TAP30_P_1409.raw
- MaxQuant (v1.5.5.1), Database: RefProteome_SPOMBE-cano_2017_10.fasta

Supplementary Figure 7b

- SAGA_161212_DHP_E_43.raw
- SAGA_161212_DHP_E_1294a.raw
- SAGA_161212_DHP_E_1294b.raw
- SAGA_161212_DHP_E_1295.raw
- MaxQuant (v1.5.5.1), Database: RefProteome_SPOMBE-cano_2016_11.fasta

Supplementary Figure 9a,b

- SAGA_171020_DHP_P_5.raw
- SAGA_171020_DHP_P_1372.raw
- SAGA_171020_DHP_P_1373.raw
- SAGA_171020_DHP_P_1375.raw
- MaxQuant (v1.5.5.1), Databases: RefProteome_SPOMBE-cano_2017_10.fasta / SAGA_3mutants_TRA1_SCHPO.fasta

- SAGA_180725_TAP40_P_1139.raw
- SAGA_180725_TAP40_P_1424-1.raw
- SAGA_180725_TAP40_P_1424-2.raw
- SAGA_180725_TAP40_P_1434-1.raw
- SAGA_180725_TAP40_P_1434-2.raw
- SAGA_180725_TAP40_P_1437.raw
- MaxQuant (v1.5.5.1), Database: RefProteome_SPOMBE-cano_2017_10.fasta
