# Supplementary material for: Chaperone-mediated ordered assembly of the SAGA and NuA4 transcription co-activator complexes in yeast
Source: Nat Commun. 2019 Nov 20;10:5237. doi: 10.1038/s41467-019-13243-w (PMC6868236; doi:10.1038/s41467-019-13243-w)
Supplement: Supplementary file 2 — Description of Additional Supplementary Files [file 41467_2019_13243_MOESM2_ESM.pdf]

Description of Additional Supplementary Files

File Name: Supplementary Data 1

Description: Summary of proteins identified by tandem affinity purification of Tti2. Shown are results of LC-MS/MS analyses of total protein mixtures from purification eluates. 'Mean FC (Log2)' numbers indicate the Log2 of the ratio [LFQ intensity in Tti2-TAP]/[LFQ intensity in no-TAP controls], averaged from four independent replicates. 'q value' numbers were calculated using a 1% permutation-based false discovery rate (FDR) in a two-tailed Student's t-test.
