# Supplementary material for: Chaperone-mediated ordered assembly of the SAGA and NuA4 transcription co-activator complexes in yeast
Source: Nat Commun. 2019 Nov 20;10:5237. doi: 10.1038/s41467-019-13243-w (PMC6868236; doi:10.1038/s41467-019-13243-w)
Supplement: Supplementary file 5 — Reporting Summary [file 41467_2019_13243_MOESM5_ESM.pdf]

nature research

Corresponding author(s): Dominique Helmlinger

Last updated by author(s): Sep 5, 2019

Reporting Summary

Nature Research wishes to improve the reproducibility of the work that we publish. This form provides structure for consistency and transparency in reporting. For further information on Nature Research policies, see Authors & Referees and the Editorial Policy Checklist.

Statistics

For all statistical analyses, confirm that the following items are present in the figure legend, table legend, main text, or Methods section.

n/a	Confirmed	
☐	☒	The exact sample size (*n*) for each experimental group/condition, given as a discrete number and unit of measurement
☐	☒	A statement on whether measurements were taken from distinct samples or whether the same sample was measured repeatedly
☐	☒	The statistical test(s) used AND whether they are one- or two-sided<br>*Only common tests should be described solely by name; describe more complex techniques in the Methods section.*
☒	☐	A description of all covariates tested
☐	☒	A description of any assumptions or corrections, such as tests of normality and adjustment for multiple comparisons
☐	☒	A full description of the statistical parameters including central tendency (e.g. means) or other basic estimates (e.g. regression coefficient) AND variation (e.g. standard deviation) or associated estimates of uncertainty (e.g. confidence intervals)
☐	☒	For null hypothesis testing, the test statistic (e.g. *F*, *t*, *r*) with confidence intervals, effect sizes, degrees of freedom and *P* value noted<br>*Give P values as exact values whenever suitable.*
☒	☐	For Bayesian analysis, information on the choice of priors and Markov chain Monte Carlo settings
☒	☐	For hierarchical and complex designs, identification of the appropriate level for tests and full reporting of outcomes
☐	☒	Estimates of effect sizes (e.g. Cohen's *d*, Pearson's *r*), indicating how they were calculated

Our web collection on statistics for biologists contains articles on many of the points above.

Software and code

Policy information about availability of computer code

Data collection	Proteomics data were collected on common commercial mass spectrometers, at the Functional Proteomics Platform (fpp.cnrs.fr) using their publicly available associated software. RNAseq data were collected on an Illumina Hiseq 4000, at Fasteris SA, using their publicly available associated software.<br>See Methods for details.
Data analysis	For RNAseq analyses, adapter sequences were trimmed from reads in the Fastq sequence files, following demultiplexing according to index barcodes. Reads were aligned using HISAT2 and counted for gene and exon features using htseq-count. Variance-mean dependence was estimated from count tables and tested for differential expression based on a negative binomial distribution, using DESeq2.<br>For proteomic analyses, raw spectra were processed using the MaxQuant environment (v1.5.0.0 or v.1.5.5.1) and Andromeda for database search with label-free quantification, match between runs and the iBAQ algorithm enabled. MS/MS spectra were matched against the UniProt reference proteome (Proteome ID UP000002485) of S. pombe (strain 972 / ATCC 24843) (Fission yeast) (release 2017_10; https://www.uniprot.org/).<br>Quantitative PCR was carried out on Stratagene Mx3005P machines, according to the MIQE guidelines.<br>Data processing and statistical analyses were performed using Microsoft Excel, Graphpad Prism (version 8.0), or R (version 3.6.0).

For manuscripts utilizing custom algorithms or software that are central to the research but not yet described in published literature, software must be made available to editors/reviewers. We strongly encourage code deposition in a community repository (e.g. GitHub). See the Nature Research guidelines for submitting code & software for further information.

Data

Policy information about availability of data

All manuscripts must include a data availability statement. This statement should provide the following information, where applicable:

- Accession codes, unique identifiers, or web links for publicly available datasets
- A list of figures that have associated raw data
- A description of any restrictions on data availability

Raw and processed RNA sequencing data are accessible from NCBI GEO under accession number GSE128448 .
Raw and processed mass spectrometry data are accessible from the ProteomeXchange Consortium via the PRIDE partner repository under the dataset identifier PXD013256.
All other data will be made available upon reasonable request.

Field-specific reporting

Please select the one below that is the best fit for your research. If you are not sure, read the appropriate sections before making your selection.

☒ Life sciences ☐ Behavioural & social sciences ☐ Ecological, evolutionary & environmental sciences

For a reference copy of the document with all sections, see nature.com/documents/nr-reporting-summary-flat.pdf

Life sciences study design

All studies must disclose on these points even when the disclosure is negative.

Sample size	Sample size was determined as described in the Methods section, whenever applicable.
Data exclusions	For RNAseq analyses, reads that did not align uniquely or not assigned to a feature were excluded.<br>For proteomic analyses, contaminants, reverse hits, non-unique peptides, only one-peptide-identified or matching-identified proteins were excluded.<br>No data were excluded from the other analyses except in case of technical failure during data acquisition.
Replication	All experiments were performed using at least three independent biological replicates, as stated in each figure legend. Technical replicates were never included in any statistical analyses. See details in the Methods section.
Randomization	No randomization was required because this study was carried our using isogenic yeast strains grown in controlled conditions.
Blinding	The investigators numbered the samples during each experiment such that data collection and analysis were performed without a priori knowledge of the genotype or culture conditions.

Reporting for specific materials, systems and methods

We require information from authors about some types of materials, experimental systems and methods used in many studies. Here, indicate whether each material, system or method listed is relevant to your study. If you are not sure if a list item applies to your research, read the appropriate section before selecting a response.

Materials & experimental systems

n/a	Involved in the study	
☐	☒	Antibodies
☒	☐	Eukaryotic cell lines
☒	☐	Palaeontology
☒	☐	Animals and other organisms
☒	☐	Human research participants
☒	☐	Clinical data

Methods

n/a	Involved in the study	
☒	☐	ChIP-seq
☒	☐	Flow cytometry
☒	☐	MRI-based neuroimaging

Antibodies

Antibodies used	peroxidase-anti-peroxidase (PAP) (P1291, Sigma)<br>anti-Calmodulin binding protein (CBP) (RCBP-45A-Z, ICLab)<br>anti-tubulin (B-5-1-2, Sigma)<br>anti-FLAG (M2, F1804, Sigma)<br>anti-MYC (9E10, Agro-Bio LC; 9E11, ab56, Abcam; and rabbit polyclonal ab9106, Abcam)<br>anti-V5 (SV5-Pk1, AbD Serotec)<br>anti-HA (16B12, Ozyme; rabbit polyclonal, ab9110, Abcam)

Validation

Antibodies were used following the manufacturers' instructions.
